# Chorea, Its Nature and Treatment

**Published:** 1856-09

**Authors:** O. C. Gibbs

**Affiliations:** Frewsburg, Chatauque County, New York


### *Chorea, its Nature and Treatment.* By O. C. Gibbs, M. D., of Frewsburg, Chatauque County, New York.

The involuntary jerking movements and imperfect subjugation of the muscular powers to the control of the will, which characterize chorea, entitle it to a place among convulsive diseases of the nervous system. It is true, it is less serious in its import than tetanus, or epilepsy, yet it is of sufficient interest to merit great attention, and has with those diseases many symptoms in common. In epilepsy there is loss of consciousness, and consequently of volition, with convulsive spasms. In tetanus there is rigid spasm, with no defect of volition or suspension of consciousness. In chorea, as in tetanus, consciousness and volition are intact; there is no deficiency of muscular energy, but that energy is exercised by a power independent of the will, and but imperfectly subject to it. In epilepsy the convulsions are paroxysmal, or intermittent; in chorea the jactitations are persistent, excepting, perhaps, during sleep, it may be for weeks or months, or, perhaps, for a lifetime. In tetanus and epilepsy the convulsive tendency is towards aggravation; in chorea, fortunately, it is generally towards amelioration and recovery.

Chorea is essentially a disease of early life, occurring mostly between the ages of five and fifteen. It is not, however, exclusively a disease of youth, as, perhaps, no period of life is exempt from its attacks. It occasionally comes on for the first time during pregnancy, in which event it usually persists until after delivery. (*See Guy's Hospital Reports*, vol. vi. Part 2. *Cases* 26 and 27.

According to the statistical observations of Rufz and others, the disease is more common in females than in males, in the proportion of about three to one. This may be accounted for on the ground of their known increased nervous susceptibility.

As we do not propose, in this brief paper, to enumerate the symptoms, diversities, and causes of chorea, we pass directly to a few observations upon its nature.

As death seldom takes place from the uncomplicated disease, the researches of the pathological anatomists have been made under unfavorable circumstances. The cerebrum, cerebellum, and spinal cord, have been repeatedly and thoroughly searched for the causative lesions upon which chorea depends. Various lesions have been observed; but, so diversified in character and so manifestly connected with complications, as to justify the conclusion that they were unconnected with the choreic symptoms. Hence, the researches of the pathological anatomist have revealed no positive light, in reference to the nature of the disease under consideration. Physiologists have attempted to locate a lesion, which, from its nature, must, perhaps, forever escape the vision of the anatomist, but so far, we believe, without uniformity of result.

The pathology of chorea, has been the subject of much controversy; yet, it is admitted by all, that the primary irritation is in the nervous system, but whether in the brain or medulla spinalis, is a question upon which the controversy hinges. Marshall Hall believes that the primary irritation is in the spinal marrow; Todd and others refer it to the brain itself. In the absence of the post mortem evidence, an opinion, in reference to the seat of the primary irritation, must be based upon the physiology of the nervous system, as productive of muscular motion, whether connected with or independent of volition. All *voluntary* movements, all acts of volition and sensation, receive their stimulus from the brain, and cannot take place without that organ. The *involuntary* movements do not of necessity involve sensation, and are performed without any participation of the will, it may be in opposition to it; they are called forth by stimuli applied to the peripheral extremities of the afferent nerves and reflected through the spinal cord. The physiology of the nervous system, as connected with volitional and automatic movements, is differently explained by different physiologists. Dr. Todd, differing somewhat from Dr. Hall and also from Dr. Muller, gives expression to his hypothesis in the following language, which we cannot do better than to quote. He says, "All the spinal and encephalic nerves, of whatever function, are implanted in the grey matter of the segments of the cerebro-spinal centre with which they are severally connected, and do not pass beyond them. The several segments of the cerebro-spinal axis are connected with each other through the continuity of the grey matter from one to another, and through the medium of commissural fibres which pass between them. Through these means, motor or sensitive impulses may be propagated from segment to segment; and stimulus conveyed to any segment from the periphery may either simultaneously affect the brain or cause a sensation, or it may be reflected upon the motor nerves of that segment, and stimulate their muscles to contract. Or both these effects may take place at the same moment, as a result of one and the same stimulus. According to this hypothesis, each segment of the cord, so long as it retains its proper commissural connexion with the brain (by commissural fibres and continuous grey matter), is part and parcel of the centre of volition as well as that of sensation, and the mind is as directly associated with each segment of the cord as it is with any portion of the encephalon. Let that commissural connexion be dissolved, and the mind will immediately lose its hold upon the cord; but the various segments of that organ may nevertheless still be acted upon by physical impulses, and may still continue to evolve the nervous force in connexion with the natural changes which may take place within." (*Physiology of the Nervous System*, p. 38.) The mechanism of *voluntary* motions, in parts supplied by spinal nerves, he thus explains:—"The impulse of volition, excited primarily in the brain, acts at the same time upon the grey matter of the cord (its anterior horn), and through it upon the anterior roots of the nerves implanted in it. This grey matter, in virtue of its association with the brain by means of the anterior pyramids, becomes a part and parcel of the organ of the will, and therefore as distinctly amenable to acts of the mind as that portion which is contained within the cranium. If we destroy the commissural connexion with the brain through the pyramidal fibres, the spinal cord ceases to take part in mental nervous actions; or, if that connexion be only partially destroyed, that portion of the cord which the injured fibres had associated with the brain, is no longer influenced by the mind. Again, if the seat of volition in the brain be diseased, the cord, or part of it, participates in the effects of the disease as far as regards voluntary actions." (P. 47.)

Dr. Todd, in his "Lumleian Lectures" for 1849, on the pathology and treatment of convulsive diseases, published in the "Medical Gazette," says, "the brain and spinal cord as the great centres of the nervous system—the great nervous battery of the body—show distinctly a division into two portions: one in which nerves are implanted; the other which has no immediate connexion with nerves, and communicates with them only through the medium of the former part." (*Lecture* 2, p. 726.) Now, if we rightly understand Dr. Todd, in the extracts given above, and we restrict the brain proper to that portion of the intracranial mass which has no connection with nerves, we can hardly see how the primary irritation in chorea can be referred to the brain itself. With Dr. Todd's view of the physiology of the nervous system and of muscular motion, the arguments of Dr. Carpenter and others, in proof of the purely cerebral origin of chorea—namely, the peculiar connection of the disordered movements with emotional excitement, and their cessation during sleep—seem to us devoid of force. The jactitating and involuntary character of the convulsive movements, performed in opposition to the will and but limitedly under its control, points to some portion of the automatic apparatus as the seat of the primary lesions in choreic convulsions. An able writer thus contradistinguishes the automatic and cerebral actions: "The sensorial ganglia, medulla oblongata, and spinal cord, are the immediate instruments of all sensorial and motor changes: by their sole and independent action are produced all those movements, which, being unprompted by the will, or even taking place in opposition to it, are said to be automatic. These automatic movements may be excited either by sensation, or by impressions which do not necessarily affect the consciousness."

"On the other hand, the cerebrum is the instrument of purely mental operations, and has no direct connexion with the external world. It receives all its stimuli to action through the sensorial ganglia; and it exerts all its influence upon the muscular system through the centres of automatic movement. Hence it may be said to be played upon by the sensorial portion of the automatic apparatus, and to play upon its motor portion."

"The will may be exerted in antagonizing, strengthening, guiding, or controlling the antomatic actions; but in every case, the muscle directly receives its power from the automatic centre; and the very same movement may be automatic, emotional, or voluntary, according as the motor influence has been excited in the centre from which the nerve proceeded, by an external stimulus acting through an afferent nerve, or by an emotional impulse transmitted downwards from the sensorial centres, or by a volition originating in the cerebrum." (*Medico-Chirurgical Review*, Vol. 5, pp. 17 and 18.) Admitting this view of the cerebral and automatic actions, and drawing the line, as above, between the brain proper and the automatic apparatus, the chain of sensory ganglia, at the base of the brain, can only be regarded as the compliment of the spinal cord, in the formation of the automatic centre on which the cerebrum is superpoised. Having then established the fact that choreic convulsions are excited by irritation, primarily within the automatic centre or reflected to it from the peripheral extremities of afferent nerves, the next inquiry is, whether that irritation is within the spinal cord, medulla oblongata, or sensorial ganglia. Choreic convulsions are occasionally succeeded by partial paralysis and hemiplegia; from this fact, Dr. Todd argues that the seat of the irritation is intra-cranial and above the decussation of the anterior pyramids. He locates the primary irritation in that portion of the sensorial ganglia which is most nearly connected with the points of implantation of the auditory and optic nerves. This opinion very nearly harmonizes with that expressed by Dr. Carpenter; and, though located within this complimental portion of the spinal cord,—the sensorial ganglia,—the topmost portion of the automatic centre, it is not within the spinal cord itself, as supposed by Dr. Hall. If additional arguments are wanted, aside from the one given, namely, the frequency of choreic hemiplegia, to prove that the primary irritation is not in the spinal cord, we may mention the frequent origination of chorea from mental causes, and also the cessation of the characteristic jactitatory movements under the influence of sleep. That the seat of irritation is not in the brain proper, derives additional confirmation from the fact that the consciousness is not affected even in the severest forms of the complaint.

Though physiologists have determined the seat of the primary irritation with a good degree of probability, yet we have no reason to expect that the pathological anatomist will ever find there, as a choreic cause, any serious organic lesion. In fact, it is next to certain, that no such lesions exist. In tetanus, whether resulting from its own natural poison or artificially induced by strychnine, there is a greater disturbance of the automatic centres than in chorea. In tetanus, unlike chorea, deaths frequently occur uncomplicated by other diseases. Yet in death resulting from tetanus, pathological anatomists have so far failed to detect any evidences of organic lesion in the cerebro-spinal centres, and we have reasons for concluding that, if the severe tonic spasms of tetanus are independent of organic lesion, the milder jactitations of chorea are no less so.

What is the nature of this irritation, seated probably in some portion of the sensorial ganglia and causative of chorea? It is said that the affection may be produced by intestinal irritation, such as worms, constipation, vitiated secretions, &c.; by fright, cold, &c.; but, is it not probable that a choreic idiosyncrasy or diathesis must previously exist? Cold and catarrh are numbered among the causes of phthisis, but a tubercular diathesis must previously exist; the local disease is the result of a constitutional cause, involving the nutritive function and stamping that function with its peculiar idiosyncrasy. Gout may succeed a debauch, but without the pre-existence of a gouty diathesis, tainting the circulating system with its poisonous products, the debauch would prove inert, so far as the development of the painful local symptoms is concerned. Though choreic convulsions result from an irritation in the sensorial portion of the automatic apparatus, yet it is highly probable that this irritation is preceded by a constitutional affection—a choreic diathesis, in some way involving the blood, resulting in depraved nutrition, and a weakened and irritable condition of the automatic centres. Dr. Todd supports this view of the nature of chorea:—upon this point, he says, "the disease is essentially one of depraved general nutrition." Dr. Simon says, "chorea confines its attacks to individuals in whom the blood development is obviously defective." (*Simon's Pathology*, p. 154.) Not only in chorea, but in all diseases where exaltation of function is evinced by the cerebro-spinal centres, as a primary nervous phenomenon, it is highly probable that the irritation in the automatic apparatus, is caused by some influence of which the blood is the vehicle. It has been thought by some, that this choreic idiosyncrasy is identical with, or similar to the rheumatic diathesis. The idea, advocated by Drs. Addison and Todd, is probably predicated upon the frequent accompaniment, in chorea, of a peculiar morbid affection of the heart, manifested by a bellows-sound, commonly mitral, and indistinguishable from that which, in rheumatism, is associated with organic lesion of the mitral valves. This idea of identity, gains some support from the fact that choreic patients are frequently the offspring of rheumatic patients, and their urine is frequently loaded with lithates, as is the case with rheumatic patients. Yet the idea of identity is not established, and may well be questioned. Perhaps we cannot better conclude our remarks upon this point than to quote from Dr. Simon. He says, "as regards the affinity between chorea and rheumatic fever, it does not, by any means, appear to me that the humoral disorder is identical for the two diseases, since they are never coincident in their occurrence; but it seems, rather, that the material which collects in the blood prior to the attack of rheumatic fever, and which, by its explosive decomposition, subsequently evolves the immense evacuations of this disease, may, while accumulating within the circulation in its original form, become capable of producing that irritation of the nervous centres which is characteriscic of chorea. (*Simon's Pathology*, p. 155.)

In the *treatment* of chorea, there is probably no remedy or set of remedies adapted to all cases; but the peculiarities of each individual case should be attentively studied, and remedial administration be made accordingly. The causes, whether predisposing or exciting, that may have given development to the disease, should be attentively sought for and removed when practicable. If a disordered condition of digestive organs, vitiated secretions, irritation of the stomach or bowels, constipation, intestinal worms, difficult dentition, &c., &c., be found connected with the disease, as a probable exciting cause, remedies should be judiciously selected for the removal of them. It is, probably, in consequence of the great variety of unhealthy conditions, that predispose to or immediately excite choreic convulsions, that so great a variety of remedies have found enthusiastic advocates with the profession in the disease under consideration. When constipation is a symptom, the bowels should be thoroughly evacuated at once, by suitable purgative medicines, and their regularity of action be secured throughout the period of subsequent treatment, by the daily administration of laxative medicines, combined with such other medicines as the peculiarities of the case may require. To allay irritation in the sensorial ganglia, counter-irritation to the back of the neck, by repeated blisters or the tartar emetic ointment, is a valuable auxiliary to the other remedies employed.

In addition to the instrumentalities mentioned, defective nutrition calls for invigorating remedies; enfeebled nervous actions, responsive to the will, require nervous stimulants and tonics; automatic excitation and jactitatory movements seem to demand nervous sedatives and antispasmodics. To give to all these indications their due significance, without undue medication, requires no little judgment and therapeutic skill. Of the invigorating remedies, iron and zinc are the most deserving of trial. Every one knows the effects of chalybeates in invigorating the nutritive functions and enriching the blood, in chlorotic conditions of the system; and it has been thought, by Dr. Golding Bird and others, that zinc exercised a similar influence on the nervous matter that iron does upon the blood. Be this as it may, the efficacy of zinc in chorea has long been established. Of the remedies directed more particularly to the nervous system, appropriate in the disease, may be mentioned musk, camphor, valerian, assafetida, cimicifuga, &c.; of these, probably, the cimicifuga is of the first importance. Its efficacy was first made known by Dr. Young, and it has since been recommended by several other eminent physicians of this country. Its *modus operandi* is not understood, but the utility of its administration, when in appropriate doses, is beyond a question. The similarity of some of the symptoms in chorea and rheumatism, finds here a counterpart in the similarity of appropriate treatment, for in chronic rheumatic pains, there is probably no remedy superior to the cimicifuga, when perseveringly given in doses sufficient to affect the nervous' system, which is evidenced by vertigo, disordered vision, headache and reduction of the circulation. Of the preparations of iron, probably the ammonio-tartrate, or the ferrocyanuret are equal to any of the others. The ammonio-tartrate possesses the advantage of perfect solubility with an agreeable taste.

The following formulæ are either of them good ones:

℞. Ferri ammonio-tart, - - ʒij.

Aquæ cinnamon, - - f. ℥iiij. ℳ.

S. A teaspoonful three or four times a day. Or,

℞. Ferri ferrocyanuret, - - gr. xv.
Extract valerianæ, - - ℈ij ss.

Make into 24 pills; S. one to be taken 3 times a day.

Of the preparations of zinc, the sulphate is the one usually employed. The following is a good formula.

℞. Zinci Sulphat. - - - ℈i.
Extract gentian. - - - ℈ij.

Make into 20 pills. One to be taken 3 times a day and gradually increased to six pills a day.

The cimicifuga may be given simultaneously with either of the formulæ above. Of the saturated tincture, two fluidrachms may be given three times a day, and it should be gradually increased until it produces sensible effects upon the nervous system, as mentioned above. These doses are appropriate for patients at or beyond the age of puberty, and should be diminished to correspond with ages below that.

The therapeutic bibliography of chorea is rich in variety; we give below a few of the more prominent instrumentalities of cure, that at different times have had enthusiastic advocates.

*Blood-letting* was recommended by Sydenham and Cullen, as well as by equally authoritative names in later times. But since it has been found that chorea is an affection connected more or less with diseased or defective nutrition, venesection has gradually, and with good reason, gone out of favor. It is now practised by but few, and then only in the acute form of the disease. The violence of the symptoms in general chorea has tempted many to use the lancet, but it has been justly observed by Dr. Todd, that the more you weaken the patient under these circumstances, the more incessant and urgent will the movements become.

*Cathartics* have been advised and strongly insisted upon in this disease by almost every writer, from the days of Sydenham to the present time. Connected as the disease nearly always is with constipation and deranged secretions, purgatives have been looked upon as an indispensable remedy, and some have considered them the only ones required. We apprehend constipation to be not so often the exciting cause of choreic convulsions as many suppose, but rather a legitimate sequence of a pre-existing choreic condition. In all disease of the brain or spinal marrow, in apoplexy and epilepsy, in coma and convulsions, constipation is usually a symptom, and we see no good reason why in chorea it should be anything else than a symptom. As such it certainly deserves attention; a cathartic should be given at first, and the bowels kept regular afterwards by suitable aperients, but purgatives should not be wholly or principally depended upon as curative agents.

*Opium* was employed by Sydenham and strongly advised by Cullen, and has since been used as a nervous sedative by many physicians, but with no very satisfactory results. The cold bath is a better sedative, and possesses the advantage of invigorating the system when judiciously and perseveringly used. The testimony of Dr. Todd bears directly upon this point. He says, "from what I have myself seen, I would say, that the greatest immediate benefit arising from the use of the cold bath is from its exciting a disposition to sleep much more decidedly than opiates, which generally do not act favorably in any of the forms of chorea." There are kinds of wakefulness, as well as of jactitatory movements, over which opium seems to have but little control, and deaths, doubtless, have occasionally occurred in the laudable attempt to produce, with this agent, results which it had not the power to accomplish. In the second volume of the American Medical Monthly, Dr. Hibben, of Brooklyn, communicates a case of death in a choreic patient, which death was to him as unexpected as it was unaccountable. When we reflect that this patient took "grain doses of sulphate of morphia, every two hours for days together; also a suppository R. morph. sulph. gr. iij., butter of cocoa, q. s." while the bowels were for six days confined, we can scarcely resist the conviction, that if death did not immediately result from medication, there was, at least, a better way to have met the indications.

*Chloroform* has been recommended as a sedative in very bad cases, and doubtless its administration is frequently of service. Dr. Barclay, in the Medical Times and Gazette, for 1853, reports a case in which arsenic, iron, cathartics, opium, &c., had failed to produce relief, but which, when the patient was much exhausted by the constant jactitations and want of sleep, was immediately relieved by the inhalation of chloroform, and ultimately cured by its occasional repetition, in connection with the regular administration of eight grain doses of quinine in suppository.

*Antimony* was advised by Breschet in as large doses as the stomach would bear without vomiting. It has been occasionally employed ever since; but seeing no indications that it is calculated to fulfil, we think it may well be dispensed with.

*Strychnia* was employed by Trousseau, and in the majority of cases with complete success. That benefit may result from the administration of this remedy we have not a doubt, but we should expect more decided results from it in chronic and hemiplegic cases than in any other.

*Electricity* has been highly commended by Drs. Bird, Addison, and others, and success claimed for it scarcely second to that of any other agent. Cases adapted to its use are probably similar to those in which strychnia is of service. This opinion is advanced with some degree of hesitancy, for it is only by more extended and scrutinizing trials that cases appropriate for either of the last two mentioned agents can be determined.

*Arsenic* has had many advocates. As a mineral tonic in chorea it is second only to iron and zinc, and in certain cachectic conditions of the system, or in patients whose healths have severely suffered from miasmatic influences, it is probably even superior to these. We presume that is in conditions like the last supposed, that quinine has in some instances proved of such marked advantage.

Many other remedies have been proposed, and some of them recommended by high authority, among which may be mentioned assafœtida, camphor, musk, oil of turpentine, nitrate of silver, iodine, hyoscyamus, hydrocyanic acid, stramonium and belladona.

